# Temperature and Nutrient Limitations Decrease Transfer of Conjugative IncP-1 Plasmid pKJK5 to Wild *Escherichia coli* Strains

**DOI:** 10.3389/fmicb.2021.656250

**Published:** 2021-07-19

**Authors:** Rebeca Pallares-Vega, Gonçalo Macedo, Michael S. M. Brouwer, Lucia Hernandez Leal, Peter van der Maas, Mark C. M. van Loosdrecht, David G. Weissbrodt, Dick Heederik, Dik Mevius, Heike Schmitt

**Affiliations:** ^1^Wetsus, European Centre of Excellence for Sustainable Water Technology, Leeuwarden, Netherlands; ^2^Department Biotechnology, Delft University of Technology, Delft, Netherlands; ^3^Department of Infectious Diseases and Immunology, Faculty of Veterinary Medicine, Utrecht University, Utrecht, Netherlands; ^4^Department of Bacteriology and Epidemiology, Wageningen Bioveterinary Research, Lelystad, Netherlands; ^5^Van Hall Larenstein, University of Applied Sciences, Leeuwarden, Netherlands; ^6^Institute for Risk Assessment Sciences, Utrecht University, Utrecht, Netherlands

**Keywords:** horizontal gene transfer, antimicrobial resistance, synthetic wastewater, soil extract agar, environmental conditions, AMR

## Abstract

Plasmid-mediated dissemination of antibiotic resistance among fecal *Enterobacteriaceae* in natural ecosystems may contribute to the persistence of antibiotic resistance genes in anthropogenically impacted environments. Plasmid transfer frequencies measured under laboratory conditions might lead to overestimation of plasmid transfer potential in natural ecosystems. This study assessed differences in the conjugative transfer of an IncP-1 (pKJK5) plasmid to three natural *Escherichia coli* strains carrying extended-spectrum beta-lactamases, by filter mating. Matings were performed under optimal laboratory conditions (rich LB medium and 37°C) and environmentally relevant temperatures (25, 15 and 9°C) or nutrient regimes mimicking environmental conditions and limitations (synthetic wastewater and soil extract). Under optimal nutrient conditions and temperature, two recipients yielded high transfer frequencies (5 × 10^–1^) while the conjugation frequency of the third strain was 1000-fold lower. Decreasing mating temperatures to psychrophilic ranges led to lower transfer frequencies, albeit all three strains conjugated under all the tested temperatures. Low nutritive media caused significant decreases in transconjugants (−3 logs for synthetic wastewater; −6 logs for soil extract), where only one of the strains was able to produce detectable transconjugants. Collectively, this study highlights that despite less-than-optimal conditions, fecal organisms may transfer plasmids in the environment, but the transfer of pKJK5 between microorganisms is limited mainly by low nutrient conditions.

## Introduction

Antimicrobial resistance (AMR) is considered as one of the most significant challenges to global public health (O’Neill, 2016). The spread of antimicrobial resistance genes (ARGs) via horizontal gene transfer (HGT) between bacteria is a growing concern because it facilitates the dissemination of resistance across a wide variety of microorganisms. Understanding the dynamics of plasmid dissemination in the environment is fundamental to contain and mitigate the AMR challenge.

Horizontal gene transfer (HGT) is an effective ecological trait that shapes bacterial evolution (Ochman et al., 2000). Conjugative plasmids are relevant vectors for HGT (Smillie et al., 2010) and dissemination of AMR (Carattoli, 2013). Gut bacteria from both animal and human origin comprise an important source of AMR-conjugative plasmids (Hu et al., 2013; Ceccarelli et al., 2019). Gut bacteria are released into the environment through manure application to agricultural soils and wastewater discharges, ultimately resulting in the introduction of their ARGs, and plasmids in the environment. Despite having limited survivability, once introduced in the environment, gut bacteria might be able to transfer their AMR determinants to the natural bacterial community. *Escherichia coli* is widely accepted as primary indicator of fecal contamination. Although most *E. coli* strains cause only mild infections, their presence is indicative of the potential presence of other more pathogenic organisms which may be relevant for human health.

Monitoring of environmental HGT remains challenging mainly due to cultivation bias [only 1% of indigenous bacteria are estimated to be cultivable (Amann et al., 1995)]. Fluorescently labeled strains and plasmids comprise a promising methodology to study horizontal gene transfer in complex environments by culture independent methods (Sørensen et al., 2005). Due to donor-recipient incompatibilities and detection limits of the methodology, the experimental design often require a compromise to guarantee the detection of transconjugants (Sørensen et al., 2005; Pinilla-Redondo et al., 2018). As a result, studies addressing environmental dissemination of AMR plasmids usually apply conditions that are optimal for bacterial transmission, namely high bacterial densities, optimal growth temperatures, and/or high nutrient availability (Bellanger et al., 2014a; Jacquiod et al., 2017). Although being relevant for specific scenarios such as mesophilic anaerobic digesters, greenhouses or wastewater in low latitude countries (Al Qarni et al., 2016; Fan et al., 2019), these settings do not reflect the usual average conditions of manured soils, water bodies and wastewater (Abis and Mara, 2006; Barrios-Hernández et al., 2020; Osińska et al., 2020). Such discrepancies in the experimental design might lead to an overestimation of plasmid transfer frequencies and dissemination potential in the environment. Therefore, better insights into how environmental parameters affect plasmid transfer are needed.

The aim of this study was to evaluate *in vitro* the role of environmental factors that could potentially hamper conjugative plasmid transfer from gut bacteria once discharged into the environment. A conjugative broad host range IncP-1plasmid (pKJK5) was used as vector. Most importantly, IncP-1 plasmids have comparatively high conjugation rates and thus allow for analysis of conjugation frequency also under suboptimal conjugation conditions. IncP-1 plasmids often carry clinically relevant ARGs (Rozwandowicz et al., 2018), are abundant in (waste)water (Pallares-Vega et al., 2021), manure (Binh et al., 2008), and soil environments (Shintani et al., 2020) and can potentially disseminate among a wide diversity of phylogenetic groups (Popowska and Krawczyk-Balska, 2013). Furthermore, IncP-1 plasmids (i.e., RP4, pB10 and pKJK5) comprise the predominant plasmids in studies addressing transfer events in environmental settings (Inoue et al., 2005; Bellanger et al., 2014b; Klümper et al., 2015; Li et al., 2018). Solid-surface filter matings were conducted to study HGT between *Escherichia coli* strains (as both donor and recipients, with animal *E. co*li strains harboring extended spectrum beta-lactamase resistance genes on known plasmid types as recipients representative of *E. coli* introduced with animal manure). The transfer was evaluated under different (i) donor-to-recipient cell proportions, (ii) mating temperatures, or (iii) nutritional compositions. The criteria to select the used conditions was based on the presumable main abiotic challenges that gut bacteria face when discharged into the environment, namely nutrient limitations and close-to psychrophilic conditions. The donor-to-recipient cell proportions were tested to assess the limit of the system while aiming for a natural proportion of donor and recipient cells in the mating. By using the same species and a broad-host-range plasmid, potential host-vector and interspecies incompatibilities were discarded as factors. *E. coli* was chosen as a model system for bacteria of public health relevance that can potentially move between anthropogenic related and natural environments, and it was hypothesized that lower temperatures and lower nutrient concentrations would limit plasmid transfer.

## Materials and Methods

### Selection and Characterization of Strains and Plasmids

Three extended-spectrum beta-lactamase (ESBL) carrying *E. coli* strains (09.54, 38.27, and 39.62) isolated from fecal samples of calves or poultry were used as recipients during the mating experiments (Table 1). These strains were part of a database from the Dutch national veterinarian institute (Wageningen Bioveterinary Research, WBVR), studying the prevalence of ESBLs in plasmids. The strains qualify for this work because of their species, diverse plasmid content, and because they had been sequenced under the scope of WBVR projects. A genetically engineered *E. coli* strain previously described by Klümper et al. (2015) was selected as donor for the broad-host-range plasmid of the incompatibility group IncP-1. The donor strain (*E. coli* K-12 MG1655:*lacI^q^-pLpp-mCherry-Km^R^*) is commonly used in dual-labeling fluorescence reporter-gene approaches coupled with fluorescence-activated cell sorting (Pinilla-Redondo et al., 2018) due to the conditionally expressible green fluorescent proteins (GFP) in its IncP-1 plasmid (pKJK5). The IncP-1 plasmid carries a kanamycin resistance determinant and lacI^q^ repressible promoter upstream the *gfpmut3* gene (Sengeløv et al., 2001; Bahl et al., 2007; Klümper et al., 2015).

**TABLE 1:** Bacterial strains of *E. coli* used as donor and recipient of broad-host-range IncP-1 plasmid, and their characteristics.

**Agent**	***ST***	**Role**	**Origin**	**Resistance profile**	**Plasmids**	**Source**
*E. coli* MG1655:lacI^q^-pLpp-mCherry-Km^R^	ST10/ST262	Donor	Laboratory strain	AMP^R^, SMX^R^, KAN^R^, mCherry pKJK5:Km^R^	pKJK5 P_A1/04/03_-gfpmut3 (IncP)	Klümper et al. (2015)
*E. coli* 09.54	ST21/ST481	Recipient	Veal calf	AMP^R^, CTX^R^, SMX^R^, TET^R^	IncK	This study
*E. coli* 38.27	ST10/ST2	Recipient	Poultry	AMP^R^, CTX^R^, SMX^R^, TET^R^	IncFI, IncH1, IncI1, p0111	This study
*E. coli* 39.62	ST101/ST88	Recipient	Poultry	AMP^R^, CTX^R^, SMX^R^, TET^R^	IncFIB/FII IncK	This study

*ST, Sequence type.*

In order to fully characterize the used strains, whole-genome sequencing using paired-end Illumina was performed, as previously described by Rozwandowicz et al. (2020). The annotation of the sequences was performed with Prokka version 1.12 (Seemann, 2014) and the corresponding sequence type a was conducted with the Multi Locus Sequence Typing online tool MLST 2.0 (Larsen et al., 2012), using the two available schemes (Wirth et al., 2006; Jaureguy et al., 2008). For typing the donor strain and relate the natural recipient strains to the donor, a reference sequence of *E. coli* MG1665 (accession number: NC_000913.3) from GenBank was used. In addition, the existence of plasmid replicons within the strains was analyzed with PlasmidFinder (Carattoli et al., 2014) applying an identity cut-off equal or greater than 98%. The annotated sequences are deposited in GenBank, BioProject PRJNA661180 under the accession no. JADPVO000000000 (09.54), JADPVP000000000 (38.27) and JADPVQ000000000 (39.62) A core and accessory genome analysis of the donor and recipient strains was conducted with Roary version 13.0 (Page et al., 2015) in Galaxy version 21.01^1^. A maximum likelihood tree based on nucleotide sequence was built with FastTree version 2.1.10 (Price et al., 2010) in Galaxy and graphic visualization of the core and accessory genome was achieved with Phandango (Hadfield et al., 2018).

**FIGURE 1:**
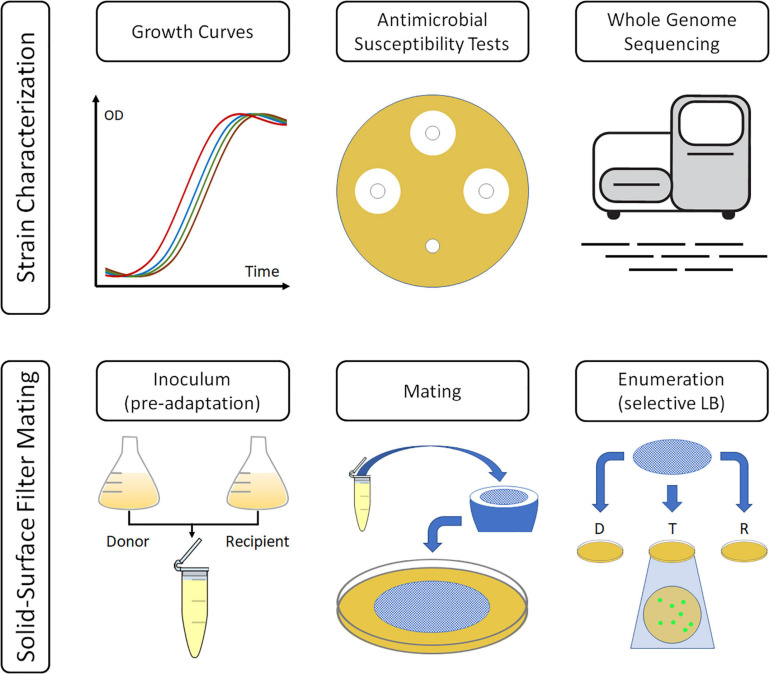
Overview of the procedure to quantify transconjugants. Donors and recipients were grown separately before being mixed, filtered, and incubated for 2 h, at different temperatures or at different media. In the end, bacteria were recovered, and enumerated, in LB containing antibiotic combinations specific for donors, recipients, or transconjugants.

To identify suitable selective conditions for the identification of transconjugants, the antimicrobial susceptibility profile for each strain was determined by disc diffusion test, according to EUCAST guidelines (EUCAST Disk Diffusion Method for Antimicrobial Susceptibility Testing – version 6.0; available at https://www.eucast.org/). The results were interpreted based on the EUCAST-defined Breakpoints tables for interpretation of MICs and zone diameters (version 8.0) and are summarized in Table 1 in Supplementary Information. Figure 1 displays this study’s schematic of the experimental design and procedure.

### Culture Media and Growth Curves

Luria-Bertani (LB), synthetic wastewater (SWW), and soil extract (SE) were used as culture media for the filter matings. Pure bacterial cultures were prepared and maintained in LB broth or plates (tryptone 10 g L^–1^, yeast extract 5 g L^–1^, sodium chloride 5 g L^–1^, and agar 15 g L^–1^) prior to the experiments, and for the selection of donor, recipients, and transconjugants after the matings, the LB plates were enriched with kanamycin (100 μg mL^–1^; Sigma Aldrich), tetracycline (16 μg mL^–1^; Sigma Aldrich), and both kanamycin and tetracycline (100 and 16 μg mL^–1^), respectively.

The SWW aimed to mimic the average conditions and nutrient proportions of conventional domestic wastewater. The composition was based on that of Boeije et al. (1999), and ISO 11733 guideline, and adjusted to a theoretical COD:N:P concentration and molar ratio close to that of Dutch wastewater (100:9.1:1.4, Supplementary Information Table 2). The SWW solution contained of 0.07 g L^–1^ urea, 0.011 g L^–1^ NH_4_Cl, 0.015 g L^–1^ peptone P (Oxoid, United Kingdom), 0.015 g L^–1^ Lab Lemco (Oxoid, United Kingdom), 0.05 g L^–1^ starch, 0.04 g L^–1^ glycerol that was sterilized by autoclaving. After sterilization, the mix was completed with 0.25 g L^–1^ sodium acetate, 0.12 g L^–1^ skimmed milk powder (Sigma Aldrich, NL), 0.05 g L^–1^ glucose, 0.025 g L^–1^ FeSO_4_, 0.005 CaCl_2_ g L^–1^, 0.025 g L^–1^ NaHCO_3_ and 0.02 g L^–1^ MgHPO_4_⋅3H_2_O, 0.016 g L^–1^ L K_3_PO_4_⋅H_2_O (unless indicated otherwise, the components were purchased at VWR, NL). These solutions were separately autoclaved, or filter sterilized prior to their aseptic addition to the final solution. SWW media was finally supplemented with the addition of 0.1% (v/v) of trace metal solution which contained 0.280 g L^–1^ NaEDTA, 0.180 g L^–1^ ZnCl_2_, 1.144 g L^–1^ H_3_BO_3_, 0.025 g L^–1^ CoCl_2_⋅6H_2_O, 0.589 g L^–1^ MnCl_2_⋅2H_2_O, 0.120 g L^–1^ CuCl_2_⋅2H_2_O, 0.068 g L^–1^ NiCl_2_⋅6H_2_O, 0.025 g L^–1^ Na_2_MoO4⋅5H_2_O, and 0.212 g L^–1^ KCr(SO_4_)_2_⋅12H_2_O. The pH was adjusted to 6.8 ± 0.1 with NaOH 1M to match the values found in wastewater [6.5 – 8.5 (Prot et al., 2020)]. When needed, agar (15 g L^–1^) was added for solid media preparation.

Soil samples for SE medium preparation were collected in the late fall of 2019, from a local dairy farm (Friesland, Netherlands) that uses the field for pasture (grassland) and had not been recently subjected to manure application. In total, 7 kg of sandy loam soil were collected from the field and homogenized. The collected soil was air-dried for 3 days and stored in 500 g zip bags at 4°C until being used. The SE media was prepared as described by Musovic et al. (2010). Briefly, 500 g of dried soil was mixed with 500 mL of demineralized water. Then, the mixture was shaken horizontally, for 3 h, and left for passive settling of the particles, for 5 h. After the 5 h, the supernatant was pipetted and autoclaved (for 15 min, at 121°C) and stored at 4°C, up to one month. The pH values were not adjusted and were kept at its original values (5.0 – 5.3), and no buffer solutions were used to maintain the pH in the different culture media because they could introduce potential nutrients (e.g., phosphate). When needed, agar was added as aforementioned.

The general chemical compositions of the LB, SWW, and SE media were determined by ion chromatography (IC), and inductively coupled plasma (ICP-OES). The determination of the chemical oxygen demand (COD), and the total nitrogen (TN) was achieved with commercially available kits (LCK 514 and LCK 338; Hach). The determination of the total organic carbon (TOC) was achieved with Shimadzu TOC-L_CPH_ analyzer. The composition of the different media used is displayed in Table 2.

**TABLE 2:** Media composition of the culture media used in the matings with either Luria-Bertani (LB), synthetic wastewater (SWW) or soil extract (SE) medium.

**(mg L^–1^)**	**LB**		**SWW**		**SE**	
**Compound**	**Mean**	**SD**	**Mean**	**SD**	**Mean**	**SD**
TOC	6,820	80	219	1.0	45	-
COD	21,450	2,450	529	37	173	1
TN	2,050	20	48	2	7	0.4
TP	151	1	7.2	0.2	4	0.0
Ca^2+^	9	1	3.6	0.0	104	1
K^+^	272	2	11.5	0.1	21	9.9
Mg^2+^	7	0.1	37.6	0.1	5	0.6
Fe^2+/3+^	0	0.0	4.5	0.0	<0.05	-
S	127	0	38	1	67	2
NH_4_^+^	60	0	6.2	0.0	<0.10	-
NO_3_**^–^**	4	0.0	<0.10	-	10	0.0
PO_4_**^3–^**	259	1	>20	-	12	0.0
SO_4_**^2–^**	96	9	11	0	191	7

*Total organic carbon (TOC), chemical oxygen demand (COD), total nitrogen (TN), total phosphorus (TP).*

To quantify the effect of the temperature change in the growth, an inoculum volume of 0.2% (final volume) of overnight culture of each strain was transferred to fresh LB, and incubated at 9, 15, 25, or 37°C. The Pathogen Modeling Program (PMP) online model (available at: https://pmp.errc.ars.usda.gov/default.aspx) was used to predict the incubation time range to measure bacterial density. To determine the effect of the nutrient composition, inoculums of 0.2% (final volume) overnight culture of each strain were transferred to SWW or SE media, and monitored up to three days. The optical density, at 600 nm (OD_600_), was measured in a UV-Vis Spectrophotometer (Shimadzu Corp). Colony forming units (CFUs) were determined after preparing 10-fold serial dilutions with saline solution (NaCl; 0.85%), plating in LB agar, and incubating at 37°C, overnight. Measurements were performed in biological triplicates.

### Solid Surface Filter Matings: Standard Conditions

Conjugation is a process that requires cell proximity and stable spatial conditions during the mating time (ca 3–5 min). Although these conditions can occur in the liquid phase, they are more likely in “surface-like” configurations (Zhong et al., 2010) occurring in soil grains, sludge flocs or biofilms. Bearing this in mind, filter mating was chosen to study the plasmid transfer.

The conjugation assays were performed by mixing 150 μL of fresh culture of the donor and recipient, and vacuum filtered through mixed-cellulose ester filters (0.45 μm; Millipore) in a Millipore filtration system. Prior to mixing, the cultures were grown for approximately 3 h in LB at 37°C to achieve a density of ca. 2 × 10^8^ CFU mL^–1^, as experimentally defined by the growth curves. After filtration, the mixed cultures were transferred to plates containing LB and cells were then incubated at 37°C. Following the incubation period, the cells were detached from the filter by vortexing in 1 mL of sterile LB broth, for 5 min. Subsequently, serial decimal dilutions were prepared in sterile saline solution, and 100 μL was spread on LB plates containing kanamycin (donors), tetracycline (recipients) and a combination of both (transconjugants). The results were observed after a 24-h incubation period (total counts), at 37°C, and another 24-h incubation period (colored colonies), at 4°C. The incubation at 4°C was performed to enhance the visualization of the GFP protein (Scott et al., 2006) and to count the green colonies, the plates were observed in a blue-light transilluminator (Safe Imager 2.0; Invitrogen). To confirm the validity of each assay, matings with only the donor or the recipient were also performed. Each mating was performed in biological triplicates on alternative days.

### Solid Surface Filter Matings: Modified Conditions

When different proportions of donor-to-recipient ratios (D/R) were tested, the donor cultures harvested until 10^8^ CFU mL^–1^ were serially diluted (10 and 100-fold) in LB and 150 μL was mixed with 150 μL recipient culture to reach the corresponding ratios D/R of 1:10 and 1:100. A total volume of 200 μL of the mixtures were then filtered, and the mating and incubation were performed as aforementioned. The approximate cell density in the filters was 8.9 × 10^6^ CFU cm^–2^. The effect of temperature in transfer frequency was assessed by following the standard condition procedure, but incubating the filters at 25, 15 and 9°C in LB plates pre-conditioned to the corresponding temperatures. To assess the influence of nutrient availability in the transfer frequency, matings conducted in SWW and SE media were compared to standard nutrient-rich media LB. For SWW matings, donor and recipient cell cultures were pre-adapted to low nutrient conditions by growing them in SWW media (1% overnight inoculum) for approximately 4 h with 180 rpm agitation until a cell density of ca. 2 × 10^8^ CFU mL^–1^ was achieved. Then, cell cultures were mixed and filtered as aforementioned in the standard conditions, and filters were placed in SWW agar plates. Plates were incubated at 37°C for 2 h. For SE matings, no pre-growth from donor nor recipients could be obtained in SE broth, as indicated by the corresponding growth curves (data not shown). Instead, late log phase LB cultures of both donor and recipients ca. 2 × 10^8^ were centrifuged and washed twice in saline solution, and the pellet was finally resuspended in 10 mL of SE broth and incubated overnight at 37°C. Before incubation, an aliquot of the resuspended cells was serially diluted in saline solution, plated in LB and incubated overnight at 37°C. Following the incubation and based on the cell counts of the suspensions, the cell density of both donor and recipient SE cultures were adjusted to approximately 2 × 10^8^ CFU mL^–1^, mixed in 1:1 ratio and filtered as indicated in the standard procedure. Filters were then placed on SE media and incubated at 37°C for 24 h. In all modified filter matings, cell recovery and subsequent plating were performed as mentioned in the standard conditions.

### Genetic Characterization of Donor, Recipient, and Transconjugants

To confirm the strain identity (donor, recipient, and transconjugants), five to ten isolates per mating were collected randomly from each of the media containing the antibiotics, and PCR was performed on the crude cell extracts. Reactions targeting the 16S rRNA gene, mCherry, and *gfpmut3* were prepared in 25-μL reactions containing PCR buffer (1x), (Invitrogen, NL) MgCl_2_ (3.0 mM), (Invitrogen, NL), dNTPs (0.2 mM) (Promega, NL), forward and reverse primers (0.4 μM; Supplementary Table 3), *Taq* polymerase (1.25 U) (Invitrogen, NL), and 1 μL of DNA. The PCR reactions were carried out in a T100 Thermal Cycler (BioRad), following similar denaturation conditions (95°C for 30 s), but specific annealing and elongation conditions (57, 55, or 60°C for 30 s; and 30 – 90 s at 72°C for the 16S rRNA, *gfpmut3*, and mCherry genes, respectively), in 30 cycles. The specificity of the PCR products was confirmed by visualization in 1.5% agarose gel stained with ethidium bromide.

### Data Analysis

One-way analysis of variance (ANOVA) was conducted to detect differences in the conjugation frequencies, between strains, temperatures, and culture media. The ANOVA tests were followed by TukeyHSD *post hoc* analysis, and homogeneity of variance was confirmed with Levene’s test. Data normality was confirmed with Shapiro-Wilk’s method, and when normality was not achieved, group comparison was performed using the equivalent non-parametric test (Kruskal-Wallis). A significance score of *p* < 0.05 was considered to be statistically relevant. These analyses were performed with R version 3.5.1 (R Core Team, 2018) and RStudio (Version 1.1.456^2^). Used software packages consisted of *reshape* (Wickham, 2007) and *tidyverse* (Wickham et al., 2019), a set of packages designed for data cleaning, trimming, and visualization; of *Rcmdr* (Fox, 2005), *PMCMRplus* (Thorsten, 2020), and *car* (Fox and Weisberg, 2019) for ANOVA and Levene’s test.

## Results

### Effect of Donor-to-Recipient (D/R) Ratios

Before the temperature and nutrients assays, the D/R ratios were tested to assess the limit of the system while aiming for a natural proportion of donor and recipient cells in the mating.

**FIGURE 2:**
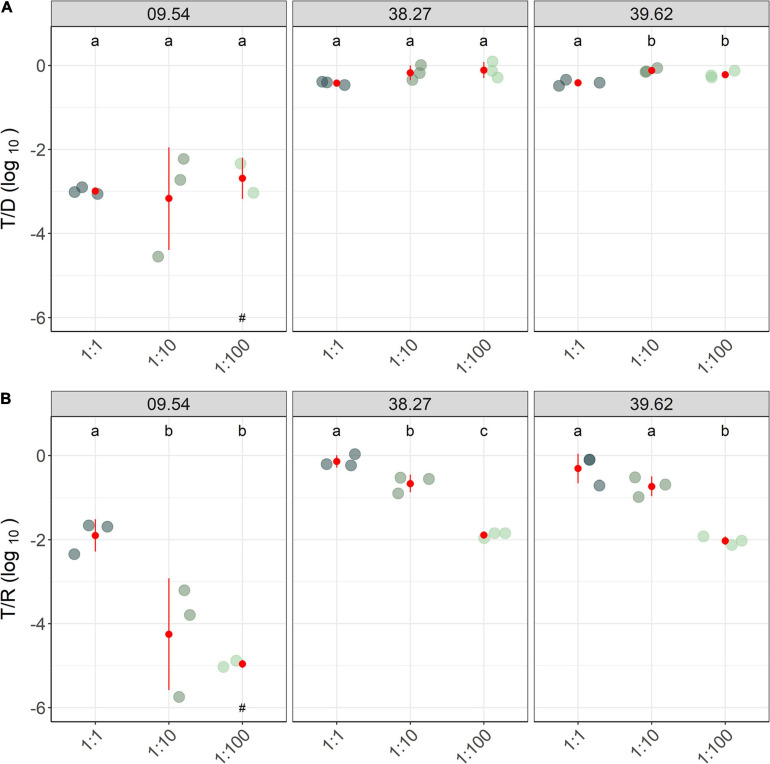
Donor-to-recipient proportions had significant effects on plasmid transfer. Depending on the indicator and strain used, the donor concentration increased or decreased, the transfer frequency. Relative counts of transconjugant-to-donor (T/D; **A**) and transconjugant-to-recipient (T/R; **B**) ratios, after 2-h matings performed at three donor-to-recipient ratios (1:1, 1:10, 1:100) are shown together with average and standard deviation values (in red). Different colors depict distinct donor-to-recipient ratios. ^a, b, c^ Indicate significantly different groups in the transfer frequency between ratios (*Post hoc* Tukey test, *p* < 0.05), and replicates with no detected transconjugants are highlighted (^#^).

Under optimal conditions and 1:1 D/R ratio (37°C and LB, 8.9 × 10^6^ CFU cm^–2^), two out of three *E. coli* strains (38.27 and 39.62) yielded high transconjugant numbers (10^9^ CFUs mL^–1^) and transfer frequency (5 × 10^–1^) of IncP-1 plasmids. On the other hand, the mating with strain 09.54 produced 10^6^ CFU mL^–1^ (transfer frequency of 10^–3^). The transfer frequency, measured as the transconjugants-to-donors ratio (T/D), resulted in a slight increase in the 1:10 and 1:100 D/R proportions in comparison with the 1:1 proportion in all strains (except for one replicate of strain 09.54; Figure 2). Contrarily, the transconjugants-to-recipients ratio (T/R) decreased with the different D/R ratios, approximately −0.7 logs and −1.8 logs in the 1:10 and 1:100 proportions, respectively (strains 38.27 and 39.62). A stronger effect of D/R was observed for strain 09.54, where the T/R decreased 1–3 logs and 3–4 logs in the 1:10 and 1:100 proportion, respectively. Similar results were found for the absolute numbers of transconjugants (Figure 1 in Supplementary information). No transconjugants were recovered for one replicate in the mating of the strains 09.54 (1:100; Figure 1). At both 1:10 and 1:100 proportions, transconjugant numbers reached approximately 10^3^ CFUs mL^–1^ for at least one of the replicates, which was close to the detection limit (10^2^ CFUs mL^–1^).

### Role of Temperature on Conjugative Transfer

Conjugation efficiency among ESBL *E. coli* strains was assessed at temperatures ranging from the optimal laboratory (37°C), room (25°C) and relevant environmental (15°C, 9°C) conditions.

**FIGURE 3:**
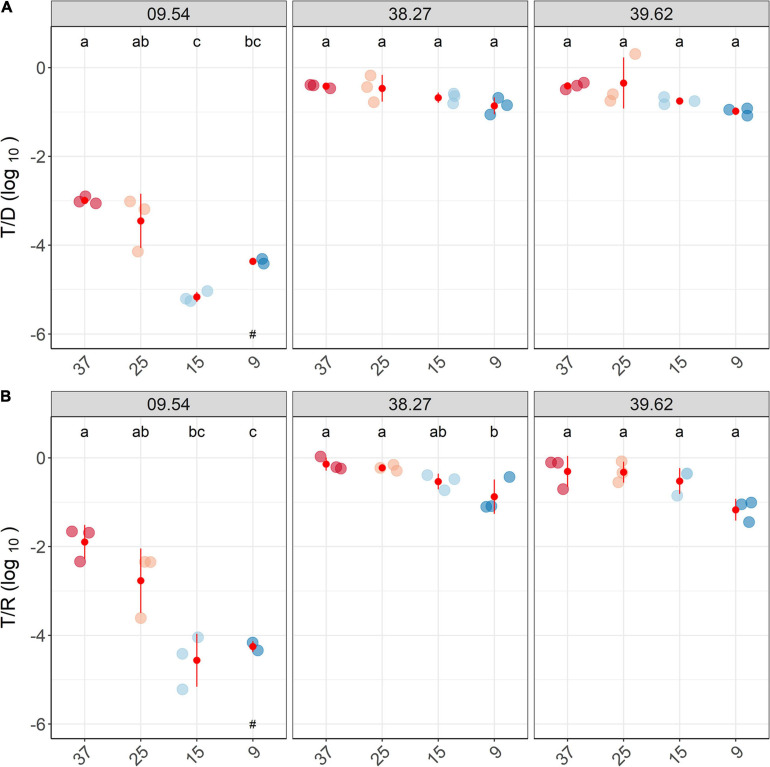
Lower temperature reduced the number of conjugation events. Relative counts of transconjugant-to-donor (T/D; **A**) and transconjugant-to-recipient (T/R; **B**) after 2h-matings performed, at diverse temperatures (37 – 9°C), are shown together with average and standard deviation values (in red). Different colors depict distinct temperatures. ^a, b, c^ Indicate significantly different groups in the transfer frequency between temperatures (*PostHoc* Tukey test, *p* < 0.05), and replicates with no detected transconjugants are highlighted (^#^).

Overall, lower temperatures significantly reduced the number of conjugation events (*p* < 0.01; Figure 3). Both T/D and T/R decreased with decreasing temperatures, with a more pronounced reduction in strain 09.54 than in the other two strains (Figure 3). The highest number of transconjugants was obtained at 37°C, and at 25°C, and the number of transconjugants decreased roughly 1 log (strains 38.27 and 39.62) or 2 logs (strain 09.54), depending on the strain. With further temperature reduction, lower transconjugant numbers were observed, and at 9°C, conjugation still occurred in all tested strains.

The lowest number of transconjugants was obtained at 9°C for strains 38.27 and 39.62. In strain 09.54, the minimum transconjugant number was already reached at 15°C and maintained at 9°C. However, higher variability among replicates was noticeable with strain 09.54 (Supplementary Figure 2), and one replicate did not yield detectable transconjugants (Supplementary Figure 2).

### Role of Nutrient Concentrations on Conjugative Transfer

Differences in plasmid transfer under diverse nutrient regimes were assessed by comparing conjugation yields and transfer frequencies between rich nutrient media (LB) and common surrogates for natural conditions such as SWW and SE media.

**FIGURE 4:**
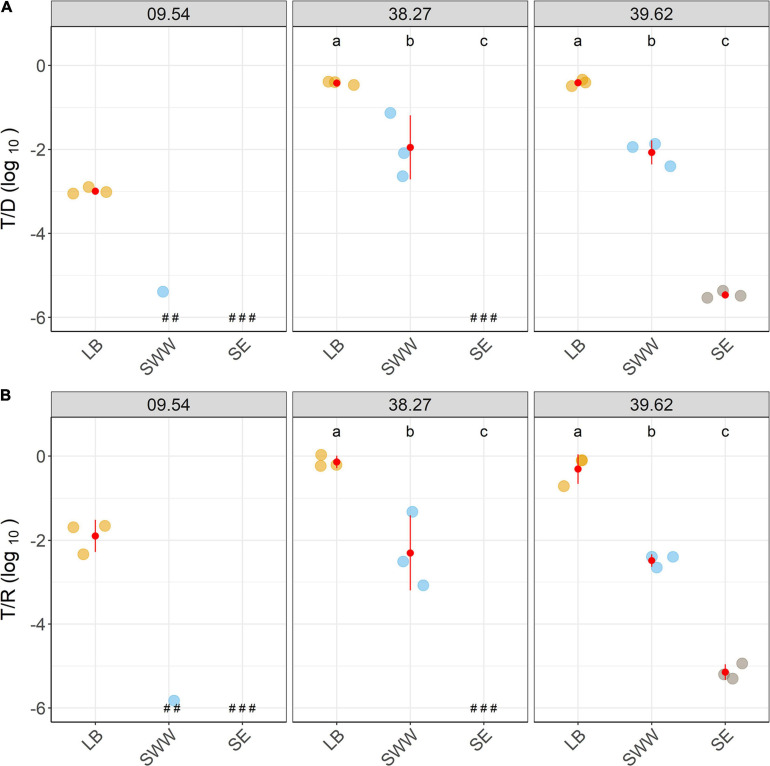
Decrease in nutrient concentration reduced conjugation events. Relative counts of transconjugant-to-donor (T/D; **A**) and transconjugant-to-recipient (T/R; **B**) after 2h-matings performed, at diverse nutrient conditions (Luria-Bertani, LB; synthetic wastewater, SWW; and soil extract, SE), are shown together with average and standard deviation values (in red). Different colors depict distinct media. ^a, b, c^ Indicate significantly different groups of transfer frequency between culture media (*PostHoc* Tukey test, *p* < 0.05), and replicates with no detected transconjugants are highlighted (^#^).

In all tested strains, the decrease in the nutrient concentration of the media resulted in a substantial decrease in conjugation events (Figure 4). In comparison with the matings performed in LB, SWW resulted in the reduction of conjugation events by roughly 2 logs. In SE, a 4-log reduction was observed for strain 39.62 (compared to LB; 4), but no transconjugants were recovered for other strains, despite several attempts.

The decline in transconjugant numbers was particularly severe for strain 09.54, which presented the lower number of transconjugants in LB. Its transconjugants were only recovered in one out of three matings performed in SWW, and when SE was used, a further decrease in the number of transconjugants was observed. While matings with strain 39.62 yielded 1.3 × 10^3^ CFUs mL^–1^ transconjugants (3 and 6 logs lower than in SWW and LB, respectively; Supplementary Figure 3), the strains 09.54 and 38.27 did not produce detectable transconjugants (Supplementary Figure 3).

## Discussion

The effects of temperature and nutrient abundance during mating of an IncP-1 plasmid were evaluated in three natural ESBL *E. coli* recipient strains by monitoring both total amounts of transconjugants and transfer frequencies. The results confirmed that psychrophilic temperatures during mating, as well as nutrient limitation, resulted in the reduction of transfer events. The decrease in the number of transconjugants was more prominent with lower nutrients than with lower temperatures.

### Transfer Efficiency Varied Across Strains

Under optimal physiological conditions for the growth of the three *E. coli* strains 09.54, 38.27, and 39.62 tested (rich LB medium, higher mesophilic temperature of 37°C), the conjugative transfer of plasmid significantly differed among the recipients. Two strains showed a high frequency of transfer (5 × 10^–1^), while the third (strain 09.54) had 2 logs less. High frequency of transfer is common among IncP-1 plasmids (Thomas and Smith, 1987), which are naturally derepressed (Bradley et al., 1980). Similar transfer frequencies (10^–2^) have been described before for the pKJK5 plasmid in soil microcosms (Musovic et al., 2006). The difference of transfer frequency among strains from the same species can relate to strain-specific characteristics or repression of silencing systems that either avoid or limit the expression of the new acquire genes in the recipient cell (Frost and Koraimann, 2010). The plasmid stability and replication depend heavily on complex coordination and synchronicity between the vector and host (Novick, 1987). In the present study, only one bacterial species (*E. coli*) was used to minimize potential genetic incompatibilities between donor and recipients. However, even when the same species are used, variable transfer frequencies are often reported. For instance, Dimitriu et al. (2019) observed a difference up to 5 orders of magnitude in the transfer frequencies of an IncF and IncP-1 among naturally co-occurring *E. coli* isolates. These significant differences are likely linked to the genetic diversity within species. Here, the accessory genes in the used strains corresponded to roughly 50% of the genomic content (Supplementary Figure 5). However, which of these accessory traits can be the cause of variation remains a matter of discussion. Dimitriu et al. (2019) found no preferential transfer among isolates sharing serotype or closely related phylogeny. Instead, they proposed that conjugal transfer was favored by clone-relationship, derived from similar restriction-modification systems. Contrarily, a recent study evaluating the transfer of ESBL plasmids among clinical *E. coli* isolates could not find such a relationship Benz et al. (2021).

In addition to host-recipient dynamics, plasmid to plasmid interactions could also affect the transfer dynamics. The stability of a newly acquired plasmid can be strongly influenced by the presence of other plasmids inside the cell (i.e., incompatibility). Here, we prevented the possible incompatibility issues by using strains with plasmids belonging to distinct Inc groups. Still, alternative effects of co-resident plasmids have been proposed recently. Enhanced transfer frequency of IncP-1 plasmids toward recipient cells hosting IncF plasmids has been observed (Gama et al., 2017). Although the mechanism of action is not entirely clear, the authors suggest that this is not a cooperative process but rather opportunistic use of the IncF transfer machinery by IncP-1 plasmids (Gama et al., 2017). In our experiments, we observed that the two strains with higher transfer frequency contained natural IncF plasmids (among others), whereas 09.54 harbored an IncK plasmid. However, further analysis would be necessary to confirm the role of co-existing plasmids in the recipient cell.

### Reducing Input of Donors Reduced Overall Transfer Frequency

A lower D/R proportion resulted in a decreased number of transconjugants, suggesting that the relative proportion of donors to recipients can limit HGT.

Receiving environmental compartments typically contain high cell densities, for instance, activated sludge usually contains between 10^9^ and 10^10^ CFU mL^–1^ (Manti et al., 2008) and topsoil (the first 10–15 cm) contain between 10^14^ and 10^15^ cells/m^3^ (Bickel and Or, 2020). However, exogenous bacteria that enter the system (potential donors) might not be as numerous. For example, assuming a soil density of 1.5, it results in having 10^8^ – 10^9^ cells/g soil, while the manure from cattle and pigs contains roughly 10^5^ *E. coli* cells/g (Schmitt et al., 2019), at least a 1,000-fold difference. This means that the proportion of potential donors is quite small considering the receiving community. This proportion may depend on multiple factors, including sewage flows or manure application rates, but it is reasonable to expect that the potential donors will be a minority in the compartment to which they were introduced.

During conjugation assays, high cell densities (8.9 × 10^6^ CFU cm^–2^) would mirror natural systems. Conversely, the use of D/R ratios lower than 1:1 (i.e., 1:10 and 1:100) would presumably reflect more accurately the conditions found in anthropogenically impacted environments. However, to observe differences in conjugation rates under varied conditions, the number of donors should be sufficient to produce a detectable amount of transconjugants with a wide margin from the limit of detection (3 to 4 logs) in the matings performed under optimal conditions. Goodman et al. (1993) and Rochelle et al. (1989) observed that a minimum of 10^4^ CFU cm^–2^ of donors and recipients were necessary to observe transconjugants. Here, conjugation occurred at donor densities as low as 10^4^ CFU cm^–2^ yielding a high amount of transconjugants (10^8^) for two of the strains (38.27 and 39.62), but not for the third one (strain 09.54). For this last strain, transconjugants were undetectable or close to the limit of detection with initial donor densities of 10^4^ or 10^5^ CFU cm^–2^ (D/R of 1:100 and 1:10, respectively). Considering that low D/R could prevent the monitoring of conjugation events for at least one of the strains, the subsequent experiments were conducted with a D/R ratio of 1:1. Similar cell densities and ratios have been previously advised to observe changes in conjugal transfer across a range of (presumably) unfavorable conditions (Fernandez-Astorga et al., 1992).

### Lower Temperature Inhibited Plasmid Transfer, but not Entirely

The highest number of transconjugants was obtained at 37°C, which is also the optimal growth temperature for *E. coli*. However, growth of donors and recipients was observed between their concentrations at the start of the experiment and in the controls (approximately 1 log, in all strains; Supplementary Figure 2). Together with growth curve data (data not shown), this suggests that, at 37°C, part of the transconjugant numbers originated from clonal expansion rather than a new transfer event. Conversely, at other temperatures, the number of transconjugants observed reflected more accurately the real number of conjugation events, as the 2-h mating time concurred with the lag phase, and, consequently, clonal expansion can assume to be negligible.

Fluctuations in temperature are known to greatly affect the growth and metabolic functions of microorganisms (Trevors et al., 2012). Yet, the effect of a wide range of temperatures on conjugative AMR-related plasmids has seldom been addressed (Bale et al., 1988; Inoue et al., 2005; Banerjee et al., 2016). Although cold conditions are predominantly found around the planet (Rodrigues and Tiedje, 2008) and in relevant environments for AMR spread (Supplementary Table 4), studies addressing the environmental dissemination of AMR plasmids in microcosms often used rather warm (>25°C) settings. Warm temperatures (25–30°C) are also common for *in vitro* studies that focus on either capturing environmental plasmids or addressing the microbial community permissiveness of a given plasmid, because high conjugation rates are required for detecting a high diversity of transconjugants (Jacquiod et al., 2017; Li et al., 2020, 2018).

Conjugation occurred at environmental temperatures (i.e., 15°C), which are average temperatures found in wastewater and soil worldwide (Supplementary Table 4), but it also occurred at 9°C. Typically, most wastewater treatment plants do not operate at temperatures below 9°C (because of nitrification failure), but in some countries, particularly northern countries, they can operate at temperatures close to 0°C (Delatolla et al., 2012; Hoang et al., 2014). The use of different strains emphasized that the effect of temperature on the transfer frequency is recipient-dependent and probably not affected just by chromosomally encoded factors, but also by resident plasmids in the recipient. The different outcomes observed between strains highlights the difficulty of inferring results that can be applicable to all putative recipient strains, even when they belong to the same species.

### Lower Nutrient Composition Hindered Conjugation

A stronger effect on the transfer frequency was observed in matings performed with lower nutrient concentrations, where the frequency of conjugation was proportional to the nutrient richness of the culture media (LB > SWW > SE). In some cases, it was not possible to recover transconjugants in SE. Some authors suggest that plasmid transfer is related to cell growth and does not occur in non-growing cells (Seoane et al., 2011; Kohyama and Suzuki, 2019), others consider that it happens after cell division and right before entering a non-growing phase (Headd and Bradford, 2020). We observed conjugation in SE media for at least one of the conjugation pairs, despite cell growth was not observed for either donor or recipients in this media.

Comparatively, the SE and SWW media used in this study contained 40 to 300-fold (SE), and 20- to 40-fold (SWW) lower basic nutrients (carbon, nitrogen and phosphorus) concentrations than the classical nutrient-rich media (LB; Table 2). Conjugation requires energy and cellular resources to occur, and thus, one could expect that low nutrient conditions would hamper plasmid transfer (Goodman et al., 1993). Interestingly, the effect of nutrient deprivation on conjugation is seldom documented. Fernandez-Astorga et al. (1992) addressed the effect of available TOC in liquid media, finding transconjugants even at 1 mg L^–1^ of TOC. Inoue et al. (2005) observed decreasing transconjugants in media with a decreasing amount of dissolved organic carbon (DOC) (6’636 to 21.6 mg L^–1^), including LB, synthetic, and real wastewater. However, in the two aforementioned studies and elsewhere (Grabow et al., 1975; O’Morchoe et al., 1988; MacDonald et al., 1992; Headd and Bradford, 2018), donor and recipient cells were pre-grown in a nutrient-rich media and then subjected to conjugation in the low nutrient media. Extra energy and nutrients stored in the cells during this pre-growth phase may allow bacteria to undergo conjugation in an earlier stage of the mating, potentially masking the effect of lower nutrition conditions on conjugation (Curtiss et al., 1969). To bypass this bias, Goodman et al. (1993) starved donors and recipients in minimal media (low amount of salts and no carbon source) prior to the conjugation. They found that, despite the lack of nutrients, conjugation occurred after the donors were starved up to 3 or 20 days, when *E. coli* or *Vibrio* sp. were the donors, respectively. In the current study, when addressing conjugal transfer in low nutrient media, cells were also pre-incubated in the corresponding low-nutrient media (SWW or SE) to avoid the influence of intracellular nutrient reservoirs.

Then again, carbon concentration is likely not the only nutrient that can limit conjugation. In their work, Inoue et al. (2005) observed that transconjugants and transfer rates were 2.5 logs higher in SWW than in 16-fold diluted LB, while both contained similar DOC content (410 mg L^–1^). Possibly, higher concentration of other nutrients (nitrogen, phosphorus or specific cations) in the SWW allowed an increase in conjugation frequencies and/or clonal expansion of the transconjugants. Pre-growth in media lacking casamino acids delayed *pili* formation after nutritional conditions are restored (Curtiss et al., 1969). As *pili* formation is protein-dependent, nitrogen-compounds are required for plasmid transfer. Despite being an essential nutrient, the role of phosphate or inorganic phosphorus deprivation in conjugation has not been explored yet. Phosphorus is known to be a limiting factor of cell growth and metabolism in oligotrophic environments (Smith and Prairie, 2004). In *E. coli*, phosphorus starvation induces a wide range of metabolic changes including cell surface modification and increase of cell adhesion characteristics (adhesins and fimbria), which could affect the interaction between cells and ultimately the conjugation rates. Finally, the concentration of other micronutrients as divalent cations might also influence conjugation. Recently, Sakuda et al. (2018) observed that the addition of divalent cations to low nutrient media (Ca^2+^ and Mg^2+^) increased the conjugation frequency of IncP-7 plasmids among *Pseudomonas* strains. Yet, the molecular mechanisms of this effect remain unclear.

Moreover, in the present study, the pH values of the different media were not maintained or adjusted, except in SWW. In SWW, the pH was adjusted to 6.8 close to the ones observed in wastewater [6.5–8.5 (Prot et al., 2020)] while the pH from SE was kept at its original value (5.0 – 5.3), which was representative of Dutch soils of this texture (Römkens and Oenema, 2004). Soil was kept at ambient pH to maintain solubility of soil nutrients. As pH can affect bacterial growth, it could have also contributed to the decrease of transconjugants in this study observed for soil. Indeed, it has been shown that pH values in this range (5.0 – 5.3) can decrease conjugation (Richaume et al., 1989), but it only resulted in a maximum of 3-fold reduction (0.5 logs) when compared to conjugation occurring at neutral pH. In the context of the present study, it is difficult to discriminate what was the effective contribution of pH in decreasing plasmid transfer in SE. However, given the several log decrease in transconjugants, it is reasonable to say that the lower nutrient content had a more important contribution in SE.

### Extrapolation of the Results and Limitations of the Study

This study addresses the influence of temperature and nutrient conditions on a specific system based on *E. coli* strains and an IncP-1 broad-host range plasmid. Probably, the impact of the factors addressed here would differ per species. Bacteria better suited to thrive under typical environmental conditions will most likely be less affected by low temperatures and nutrient conditions, as observed by a longer ability (+13 days) for conjugal transfer when using pre-starved *Vibrio* spp. as donor instead of *E. coli* (Goodman et al., 1993). In addition, the plasmid characteristics (e.g., size, incompatibility group) obviously determine absolute transfer rates. Thus, further research addressing other combinations of donors-recipients will be desirable.

## Conclusion

When moving from laboratory conditions to environmentally relevant conditions for soils and WWTPs, both lower temperature and lower nutrient concentrations showed to reduce conjugal transfer of an IncP-1 plasmid significantly. The effect lower nutrient concentrations on the number of transconjugants was stronger than the effect of lower temperatures. While nutritional conditions appear critical, the role of single nutrients, such as nitrogen and phosphorus, is not entirely clear and deserves further follow-up research. Furthermore, the transfer potential was recipient-dependent and varied within ESBL *E. coli* strains of the same species.

To conclude, although abiotic factors can hamper plasmid transfer, measurable conjugation between *E. coli* still occurred under conditions that mimicked those commonly found in the wastewater and soil environment (9 – 25°C). Despite conjugation being observed between strains of the same species, this study shows that fecal indicator bacteria were capable of donating an IncP-1 plasmid in less-than-optimal contexts, and consequently, can be a source of transferable AMR traits once they reach the environment.

## Data Availability Statement

The datasets presented in this study can be found in online repositories. The names of the repository/repositories and accession number(s) can be found in the article/Supplementary material.

## Author Contributions

RP-V, GM, LH, and HS conceived and designed the study. RP-V and GM performed the experiments and analyzed the data. MB and RP-V performed the analysis of the next-generation sequencing data. MB, LH, DM, DH, ML, DW, and HS supervised the study. RP-V and GM wrote the manuscript. MB, LH, PM, ML, DW, DM, DH, and HS reviewed and edited the manuscript. All authors read and approved the final manuscript.

## Conflict of Interest

The authors declare that the research was conducted in the absence of any commercial or financial relationships that could be construed as a potential conflict of interest.

## Footnotes

1 https://usegalaxy.eu/

2 https://www.rstudio.com/
