# Supplementary material for: Temperature and Nutrient Limitations Decrease Transfer of Conjugative IncP-1 Plasmid pKJK5 to Wild Escherichia coli Strains
Source: Front Microbiol. 2021 Jul 19;12:656250. doi: 10.3389/fmicb.2021.656250 (PMC8326584; doi:10.3389/fmicb.2021.656250)
Supplement: Supplementary file 1 [file Data_Sheet_1.docx]

Supplementary Material

Temperature and nutrient limitations decrease transfer of conjugative IncP-1 plasmid pKJK5 to wild *Escherichia coli* strains

Rebeca Pallares-Vega^1,2^†, Gonçalo Macedo^1,3^†, Michael SM Brouwer^4^, Lucia Hernandez Leal^1^, Peter van der Maas^5^, Mark CM van Loosdrecht^2*^, David G Weissbrodt^2^, Dick Heederik^6^, Dik Mevius^3,4^, Heike Schmitt^1,3,6^

^1^ Wetsus, European Centre of Excellence for Sustainable Water Technology, Oostergoweg 9, 8911 MA Leeuwarden, The Netherlands

^2^ Dept. Biotechnology, Delft University of Technology, Van der Maasweg 9, 2629, HZ Delft, the Netherlands

^3^ Department of Infectious Diseases and Immunology, Faculty of Veterinary Medicine, Utrecht University, Yalelaan 1, 3584 CL Utrecht, The Netherlands

^4^ Department of Bacteriology and Epidemiology, Wageningen Bioveterinary Research, Houtribweg 39, 8221 RA Lelystad, The Netherlands

^5^ Van Hall Larenstein, University of Applied Sciences, Agora 1, 8901 BV Leeuwarden, The Netherlands

^6^ Institute for Risk Assessment Sciences, Utrecht University, Yalelaan 2, 3584 CM Utrecht, The Netherlands

*** Correspondence:**Mark van Loosdrecht
[M.C.M.vanLoosdrecht@tudelft.nl](mailto:M.C.M.vanLoosdrecht@tudelft.nl)

† These authors have contributed equally to this work and share first authorship.

The Supplementary Materials include:

- Tables S1-S4
- Figures S1-S3
- References

**Table S1.** Antibiotic susceptibility determined by disc diffusion test, according to EUCAST guidelines. Strains are classified in Resistant (R), Susceptible (S) or Intermediate resistance (I).

| Strain | Ampicillin | Cefotaxime | Ciprofloxacin | Gentamicin | Sulfamethoxazole | Tetracycline | Kanamycin | Rifampicin |
| --- | --- | --- | --- | --- | --- | --- | --- | --- |
|  | AMP (10 µg) | CTX (5 µg) | CIP (5 µg) | GEN (10 µg) | SMX (25 µg) | TET (30 µg) | KN (30 µg) | RF (5 µg) |
| *E. coli* MG1655::*lacI^q^-pLpp-mCherry-Km^R^ /* pKJK5::*P_A1/04/03_-gfpmut3* | R | S | S | S | R | S | R | R |
| 09.54 | R | R | S | S | R | R | I | R |
| 39.62 | R | R | S | S | R | R | S | R |
| 38.27 | R | R | S | S | R | R | I | R |

**Table S2.** Average annual concentrations of organic matter (COD, BOD), total nitrogen (TN) and total phosphorus (TP) in the influent wastewater of Dutch WWTPs between 2000-2018. Source CBS: <https://opendata.cbs.nl/statline/#/CBS/nl/dataset/7477/table?dl=3DD6>. **Abbreviations:** pe– population equivalents, COD – Chemical Oxygen Demand; BOD – Biological Oxygen Demand.

| Year | Number of WWTPs | Total capacity (x1.000 pe) | COD (mg L^-1^) | BOD (mg L^-1^) | TN (mg L^-1^) | TP (mg L^-1^) |
| --- | --- | --- | --- | --- | --- | --- |
| 2000 | 391 | na | 470 | 180 | 43 | 7 |
| 2001 | 384 | na | 461 | 175 | 42 | 7 |
| 2002 | 378 | na | 477 | 185 | 44 | 7 |
| 2003 | 378 | na | 550 | 213 | 51 | 9 |
| 2004 | 375 | na | 506 | 194 | 46 | 8 |
| 2005 | 368 | na | 525 | 198 | 48 | 8 |
| 2006 | 363 | na | 520 | 196 | 48 | 8 |
| 2007 | 356 | na | 471 | 174 | 44 | 7 |
| 2008 | 351 | na | 503 | 192 | 47 | 8 |
| 2009 | 351 | na | 536 | 208 | 49 | 8 |
| 2010 | 349 | 30,365 | 513 | 200 | 46 | 7 |
| 2011 | 346 | 30,383 | 526 | 206 | 48 | 7 |
| 2012 | 343 | 30,358 | 505 | 199 | 46 | 7 |
| 2013 | 341 | 30,364 | 520 | 209 | 48 | 7 |
| 2014 | 337 | 30,237 | 548 | 218 | 50 | 7 |
| 2015 | 334 | 30,246 | 516 | 209 | 45 | 7 |
| 2016 | 327 | 30,122 | 541 | 225 | 49 | 7 |
| 2017 | 326 | 29,904 | 546 | 232 | 49 | 7 |
| 2018 | 323 | 29,942 | 593 | 248 | 54 | 7 |
|  |  | **Average** | 517 | 203 | 47 | 7.4 |
|  |  | **SD** | 32 | 19 | 3 | 0.6 |
|  |  | **COD:N:P ratio** | 100 |  | 9.1 | 1.4 |

**Table S3.** Primers used for PCR confirmation of strains.

| Target | Primer name | Sequence (5´- 3´) | Amplicon size (bp) | Reference |
| --- | --- | --- | --- | --- |
| 16S rRNA | 27F | AGA GTT TGA TCC TGG CTC AG | 1465 | (Frank et al., 2008) |
|  | 1492R | GGT TAC CTT GTT ACG ACT T |  |  |
| *gfpmut3* | q_GFPmut3-FW | TCG GTT ATG GTG TTC AAT GC | 146 | (Norman et al., 2014) |
|  | q_GFPmut3-RV | GAC TTC AGC ACG TGT CTT GTA G |  |  |
| *mCherry* | q_mCherry-FW | CCC CGT AAT GCA GAA GAA GA | 99 | Eurofins Genomics  (Vidgren and Gibson, 2018) |
|  | q_mCherry-RV | TTC AGC CTC TGC TTG ATC TC |  |  |

**Table S4.** Temperature range in wastewater and soil in cold countries. Acronyms: WW: Wastewater. NA: Not available

| Country | Sampling period | |  | Temperature (°C) | |  | Sample | | Reference |
| --- | --- | --- | --- | --- | --- | --- | --- | --- | --- |
|  | Year | Months |  | Min | Max |  | Matrix | Type |  |
| Austria | DS | March April- June -July |  | 7.8 | 15.5 |  | WW | Sewer | (Kretschmer et al., 2016) |
| China | 2013  2014 | January-April-June-October |  | 15 | 30 |  | WW | NA | (Liu et al., 2016) |
| Finland | 2010  2011 | Each season |  | 9 | 18 |  | WW | NA | (Karkman et al., 2016) |
| The Netherlands | 2017  2018 | December to May |  | 9 | 15 |  | WW | NA | (Barrios-Hernández et al., 2020) |
| The Netherlands | 2009-2011 |  |  | 9 | 20 |  | WW | NA | (Krzeminski et al., 2012) |
| Poland | 2015 | January-April-July-October |  | 8.7 | 20.9 |  | WW | Influent | (Osińska et al., 2017) |
|  |  |  |  | 9.5 | 21.3 |  | WW | Effluent |  |
| USA  (Minnesota) | - | - |  | 10 | 20 |  | WW | Influent | (Johnston et al., 2019) |
| Czech Republic | 1961-2000 | Monthly |  | -0.5 | 21.2 |  | Soil | -5 cm | (Pokladníková et al., 2008) |
|  |  |  |  | 0.4 | 19.9 |  | Soil | -20 cm |  |
| China  (north) |  | Monthly average |  | -2 | 16 |  | Soil |  | (Zhou et al., 2015) |
| Germany  (Leibniz) | 1894-2019 | Monthly average |  | -7 | 35 |  | Soil | -5 cm | 1 |
|  |  |  |  | -9 | 27 |  | Soil | -20 cm |  |
| The Netherlands | 2006-2020 | Monthly average |  | 1 | 21 |  | Soil | -5cm | 2 |
|  |  |  |  | 1 | 21 |  | Soil | -20 cm |  |

1 <https://www.pik-potsdam.de/services/climate-weather-potsdam/climate-diagrams/ground-temperature>

2 <http://projects.knmi.nl/cabauw/insitu/observations/soiltemp/>


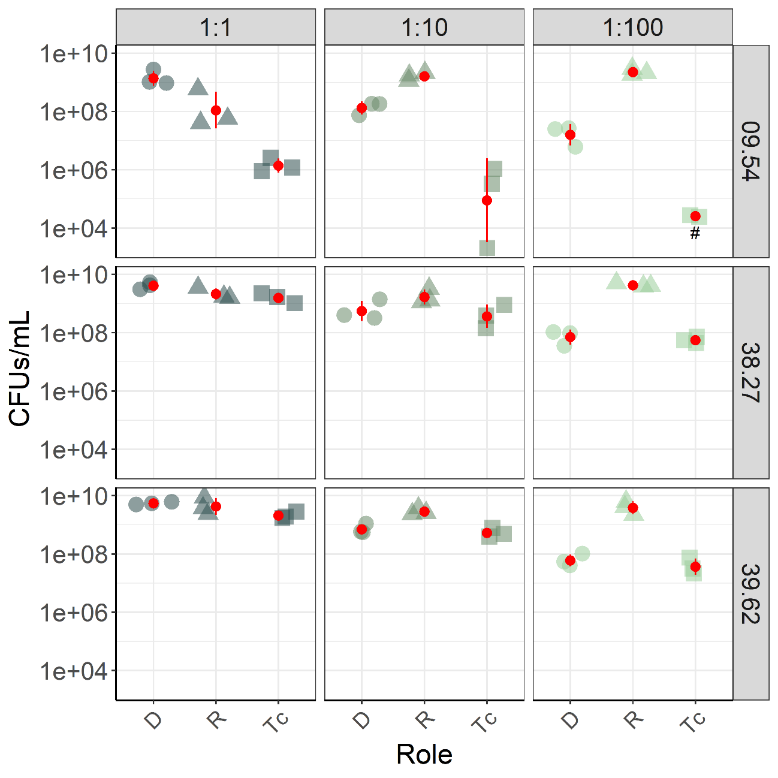


**Figure S1.** Absolute counts of donors (D), recipients (R), and transconjugants (Tc) after 2h-matings performed with different D/R proportions (1:1, 1:10, 1:100) for each strain. The averages and standard deviations of the matings are displayed in red. (#) indicate replicates without detectable transconjugants. Limit of detection was 10^2^ CFUs mL^-1^


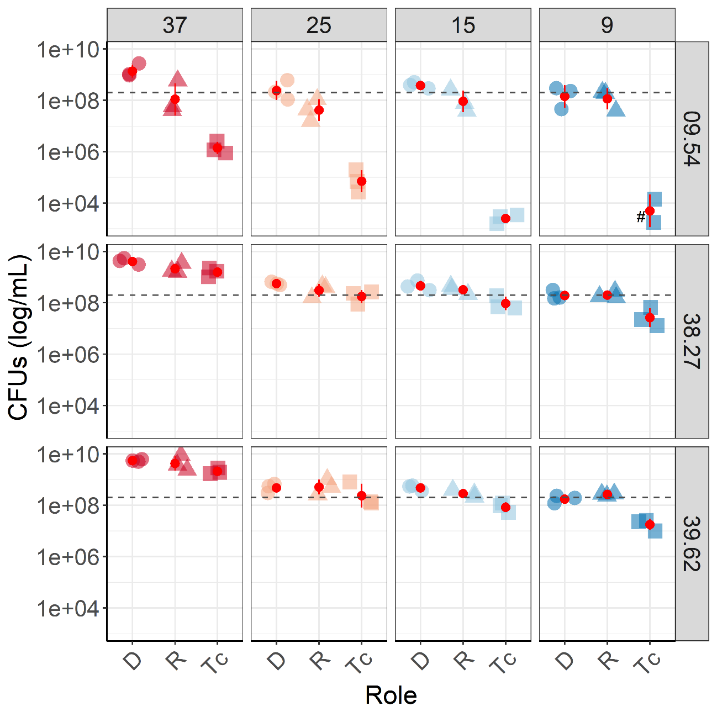


**Figure S2.** Absolute counts of donors (D), recipients (R), and transconjugants (Tc) after 2h-matings under diverse temperatures. The grey dashed line (2 x 10^8^) indicates the approximate original number of cells in the beginning of the mating. (#) indicate replicates without detectable transconjugants. Limit of detection was 10^2^ CFUs mL^-1^


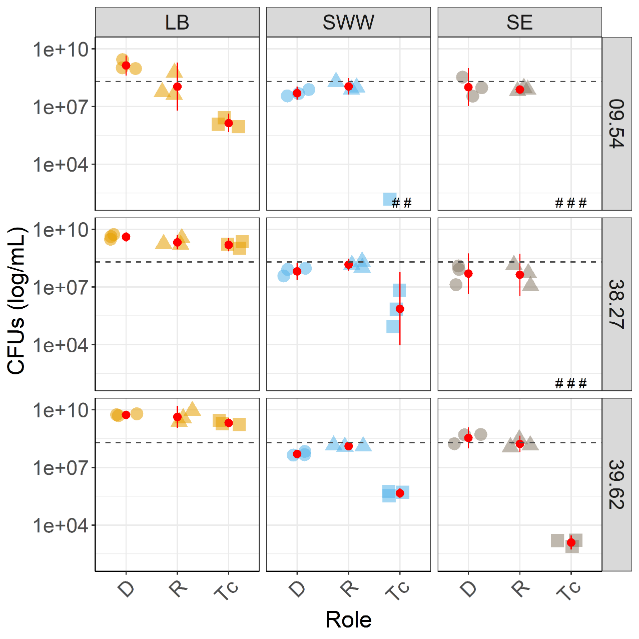


**Figure S3.** Absolute counts of donor (D), recipient (R) and transconjugants (Tc) after 2h mating in LB, Synthetic wastewater (SWW) and Soil Extract (SE) media. The grey dashed line (2 x 10^8^) indicates the approximate original number of cells in the beginning of the mating. (#) stands for the replicates with no detected transconjugants. Limit of detection was 10^2^ CFUs mL^-1^


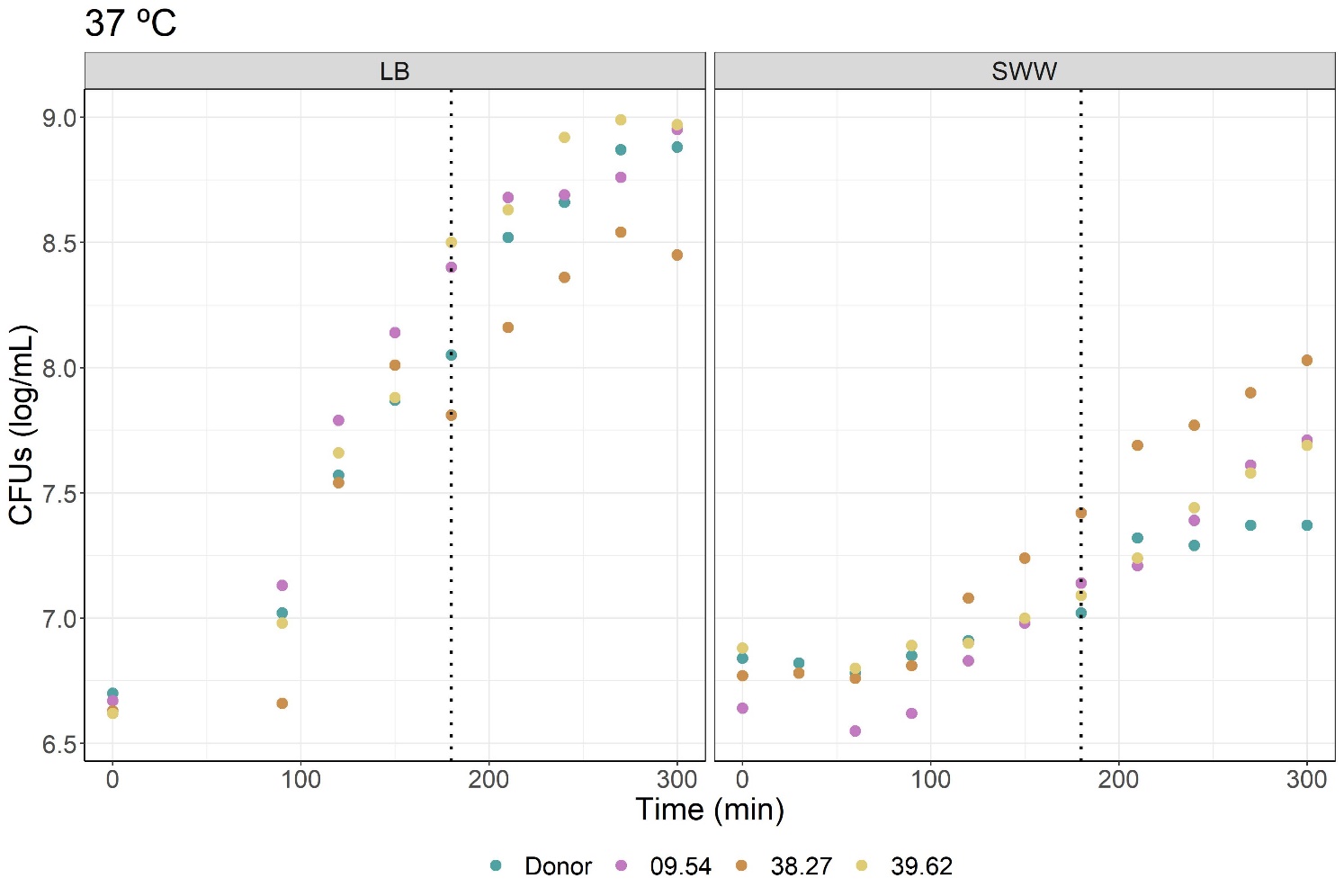


Figure S4: Growth curves in LB and synthetic wastewater (SWW) at 37ºC, with 0.2% of o/n inoculum performed for the four tested strains: the donor and wild type recipients (09.54, 38.27 and 39.62). No growth curve is presented for soil extract (SE) because no growth was observed in SE. Vertical dashed line indicates 180 min (3h), usual time for incubation before mating.


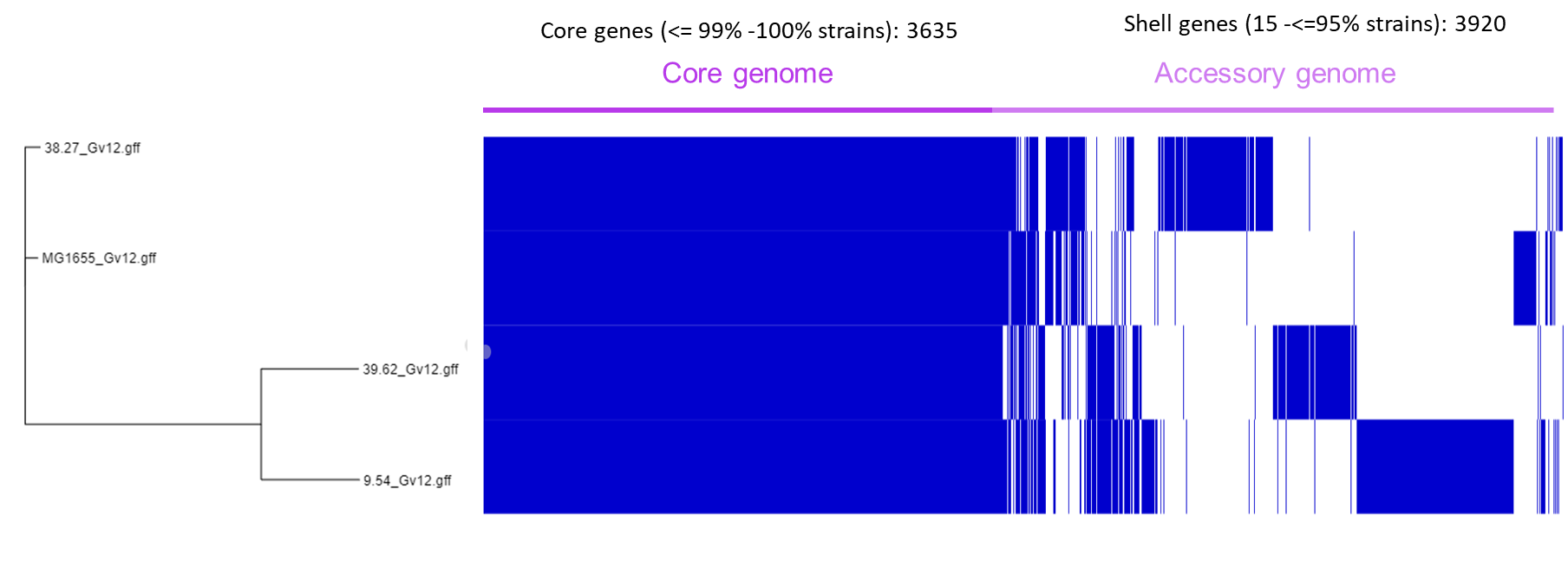


Figure S5: Core and accessory genome of the four strains used in this study. Since the sequence of the donor strain was not available, a RefSeq sequence of the same *E. coli* strain used as donor (*E. coli* MG1655) was retrieved from GenBank for the analysis (accession number NC_000913.3).

**References**

Barrios-Hernández, M.L., Pronk, M., Garcia, H., Boersma, A., Brdjanovic, D., van Loosdrecht, M.C.M., Hooijmans, C.M., 2020. Removal of bacterial and viral indicator organisms in full-scale aerobic granular sludge and conventional activated sludge systems. Water Res. X 6. https://doi.org/10.1016/j.wroa.2019.100040

Frank, J.A., Reich, C.I., Sharma, S., Weisbaum, J.S., Wilson, B.A., Olsen, G.J., 2008. Critical evaluation of two primers commonly used for amplification of bacterial 16S rRNA genes. Appl. Environ. Microbiol. 74, 2461–2470. https://doi.org/10.1128/AEM.02272-07

Johnston, J., LaPara, T., Behrens, S., 2019. Composition and Dynamics of the Activated Sludge Microbiome during Seasonal Nitrification Failure. Sci. Rep. 9, 4565. https://doi.org/10.1038/s41598-019-40872-4

Karkman, A., Johnson, T.A., Lyra, C., Stedtfeld, R.D., Tamminen, M., Tiedje, J.M., Virta, M., 2016. High-throughput quantification of antibiotic resistance genes from an urban wastewater treatment plant. FEMS Microbiol. Ecol. 92, fiw014. https://doi.org/10.1093/femsec/fiw014

Kretschmer, F., Simperler, L., Ertl, T., 2016. Analysing wastewater temperature development in a sewer system as a basis for the evaluation of wastewater heat recovery potentials. Energy Build. 128, 639–648. https://doi.org/10.1016/j.enbuild.2016.07.024

Krzeminski, P., Iglesias-Obelleiro, A., Madebo, G., Garrido, J.M., van der Graaf, J.H.J.M., van Lier, J.B., 2012. Impact of temperature on raw wastewater composition and activated sludge filterability in full-scale MBR systems for municipal sewage treatment. J. Memb. Sci. 423–424, 348–361. https://doi.org/10.1016/j.memsci.2012.08.032

Liu, T., Liu, S., Zheng, M., Chen, Q., Ni, J., 2016. Performance assessment of full-scale wastewater treatment plants based on seasonal variability of microbial communities via high-throughput sequencing. PLoS One 11. https://doi.org/10.1371/journal.pone.0152998

Norman, A., Riber, L., Luo, W., Li, L.L., Hansen, L.H., Sørensen, S.J., 2014. An Improved Method for Including Upper Size Range Plasmids in Metamobilomes. PLoS One 9, e104405. https://doi.org/10.1371/journal.pone.0104405

Osińska, A., Korzeniewska, E., Harnisz, M., Niestępski, S., 2017. The prevalence and characterization of antibiotic-resistant and virulent Escherichia coli strains in the municipal wastewater system and their environmental fate. Sci. Total Environ. 577, 367–375. https://doi.org/10.1016/j.scitotenv.2016.10.203

Pokladníková, H., Rožnovský, J., Středa, T., 2008. Evaluation of soil temperatures at agroclimatological station pohořelice. Soil Water Res. 3, 223–230. https://doi.org/10.17221/2092-swr

Vidgren, V., Gibson, B., 2018. Trans-regulation and localization of orthologous maltose transporters in the interspecies lager yeast hybrid. FEMS Yeast Res. 18, 1–14. https://doi.org/10.1093/femsyr/foy065

Zhou, Z., Xu, M., Kang, F., Sun, O.J., 2015. Maximum temperature accounts for annual soil CO2 efflux in temperate forests of Northern China. Sci. Rep. 5, 1–10. https://doi.org/10.1038/srep12142
